# Dolutegravir(DTG, S/GSK1349572) combined with other ARTs is superior to RAL- or EFV-based regimens for treatment of HIV-1 infection: a meta-analysis of randomized controlled trials

**DOI:** 10.1186/s12981-016-0115-x

**Published:** 2016-09-08

**Authors:** Junjun Jiang, Xi Xu, Wenqin Guo, Jinming Su, Jiegang Huang, Bingyu Liang, Hui Chen, Ning Zang, Yanyan Liao, Li Ye, Hao Liang

**Affiliations:** 1 Guangxi Key Laboratory of AIDS Prevention and Treatment & Guangxi Universities Key Laboratory of Prevention and Control of Highly Prevalent Disease, School of Public Health, Guangxi Medical University, Nanning, 530021 Guangxi China; 2 Guangxi Center for Disease Control and Prevention, Nanning, 530028 Guangxi China; 3 Geriatrics Digestion Department of Internal Medicine, The First Affiliated Hospital of GuangXi Medical University, Nanning, 530021 Guangxi China; 4 Guangxi Collaborative Innovation Center for Biomedicine, Life Sciences Institute, Guangxi Medical University, Nanning, 530021 Guangxi China

**Keywords:** DTG, HIV/AIDS, Meta-analysis, Efficacy, Safety

## Abstract

**Background:**

The first-generation integrase inhibitors (INIs) raltegravir (RAL) and elvitegravir (EVG) have shown efficacy against HIV infection, but they have the limitations of once-more daily dosing and extensive cross-resistance. Dolutegravir (DTG, S/GSK1349572), a second-generation drug that overcomes such shortcomings, is under spotlight. The purpose of this study is to review the evidence for DTG use in clinical settings, including its efficacy and safety.

**Methods:**

PubMed, EMbase, Ovid, Web of Science, Science Direct, and related websites were screened from establishment until July 2013, and scientific meeting proceedings were manually searched. Two reviewers independently screened 118 citations repeatedly to identify randomized controlled trials comparing the efficacy and safety of DTG-based regimen with those of RAL- or elvitegravir-based regimens. Using the selected studies with comparable outcome measures and indications, we performed a meta-analysis based on modified intention-to-treat (mITT), on-treatment (OT), and as-treated (AT) virological outcome data. Independent data extraction and quality assessment were conducted.

**Results:**

Four unique studies were included with the use of DTG in antiretroviral therapy-naive patients. In therapy-naive patients, DTG combined with abacavir/lamivudine (ABC/3TC) or tenofovir/emtricitabine (TDF/FTC) resulted in a significantly better virological outcome with a mITT relative risk (RR)of 1.07 (95 % confidence interval (95 % CI 1.03–1.12). Evidence further supported use of DTG had a better virological suppression in the 50 mg once daily group (mITT RR 1.07; 95 % CI 1.03–1.12) as well as in the sub-analysis in dolutegravir/efavirenz(DTG/EFV) and dolutegravir/raltegravir (DTG/RAL) groups (RR 1.09, 95 % CI 1.03–1.15; RR 1.06, 95 % CI 0.98–1.15, respectively). In the matter of safety of DTG-based regimen, the risk of any event was RR 0.98 (95 % CI 0.94–1.01), the risk of serious adverse events (AEs) was RR 0.84 (95 % CI 0.62–1.15), and the risk of drug-related serious AEs was RR 0.33 (95 % CI 0.13–0.79).

**Conclusion:**

In general, DTG 50 mg given once daily combined with an active background drug is a better choice in terms of both efficacy and safety.

## Background

Since acquired immune deficiency syndrome (AIDS) was first identified as a serious communicable disease in 1981, an estimated 35.3 (32.2–38.8) million people were living with HIV in 2012 [1]. Stable integration of the reverse-transcribed viral genome into host chromatin forms a significant mechanism during HIV infection. Integrase inhibitors (INIs) are a class of antiretroviral drugs targeting the strand transfer reaction during the integration process. It is active against HIV-1 strains that are resistant to nucleoside or nucleoside reverse transcriptase inhibitors (NRTIs), non-nucleoside reverse transcriptase inhibitors (NNRTIs), and protease inhibitors [2]. Unlike other enzymes that exist in viruses and humans, integrase enzymes are absent in mammalian cells. Therefore, blockade of integrase is highly specific to viruses and is associated with low toxicity [3]. The first-generation INIs raltegravir (RAL) and elvitegravir (EVG) have shown high efficacy against HIV in treatment-naive and treatment-experienced individuals [4–10]. Nevertheless, RAL must be taken twice daily, while EVG requires a pharmacokinetic booster such as ritonavir or cobicistat [11–13]. Moreover, there is extensive cross-resistance between RAL and EVG [14]. Dolutegravir (DTG, S/GSK1349572), a new INI drug without such shortcomings, is under spotlight. It is an effective inhibitor of HIV integrase and HIV replication in cell culture assays even at low concentrations of nanomolar level [11]. Pharmacokinetic studies in people have also shown DTG has a long plasma half-life without the need for a booster [15]. Furthermore, significant reductions in plasma HIV-1 viral load from baseline were observed for all DTG regimen groups compared with placebo (*p* < 0.001), with a mean decrease of 1.51–2.46 log10 copies/mL [14]. Finally, results from in vitro passage experiments showed its potential for a higher barrier to drug resistance compared with RAL and EVG [12]. The VIKING study has suggested that DTG is active against HIV-1 strains harbouring major integrase inhibitor resistance mutations selected by both RAL and EVG [15–18]. Taken together, these studies show that DTG has low cross-resistance, with the potential for a higher barrier to resistance than other integrase inhibitors.

Combination therapy is the recognised care for HIV-infected patients. Potential interaction between S/GSK1349572 and other antiretroviral drugs has been evaluated in previous studies. Most antiretroviral and other drugs often prescribed for people with HIV infection do not have clinically relevant interaction with DTG, according to results of several studies [19]. To assess the efficacy and safety of DTG-based regimens, randomized controlled trials (RCTs) were designed to compare DTG-based regimen with the currently approved combination therapy. Some studies found that a switch to a DTG-based therapy was not superior to the approved combination therapy [20, 21]. However, in other studies, statistical superiority of a DTG-based therapy over the approved combination therapy was also reported [22, 23]. Thus, the results of these studies are still inconclusive. This issue sparks our interesting to pool samples from RCTs and to get an enlarged sample size, which can make the evaluation more reliable. In this study, a meta-analysis of current evidence was conducted to assess the safety and efficacy of DTG in combination with other antiretroviral drugs.

## Methods

### Data sources and searches

We followed a protocol using the methodological approaches outlined in the Agency for Healthcare Research for Effectiveness and Comparative Effectiveness Reviews [24]. The systematic review included both published studies and reports from scientific meeting proceedings. Two independent reviewers screened the titles and abstracts of eligible studies. The literature search was conducted in PubMed, EMbase, Ovid, Web of Science, Science Direct database from their establishments to July 2013, and limited to English publications. The key words used were as follows: “HIV”; “dolutegravir”; “DTG”; “S/GSK1349572”; or “integrase inhibitor”. At the same time, if we found any related documents, we went back to its references for further searching.

### Study selection

Inclusion and exclusion criteria were as follows:

1. We included RCTs comparing DTG-based regimen with RAL- or elvitegravir-based regimens in efficacy and safety. and excluded studies on experimental drugs, reviews, and technical papers.
2. Language restriction was set to English.
3. We included trials with the same or a similar study design, prior to those with the same randomized period. We excluded studies on pharmacokinetics and dynamics. When the related studies by the same authors were found, we chose the study with the biggest sample size and most typical samples.
4. We included original research papers and abstracts of clinical trials on the use of DTG in HIV-positive patients and excluded in vitro and animal studies.

**Fig. 1:**
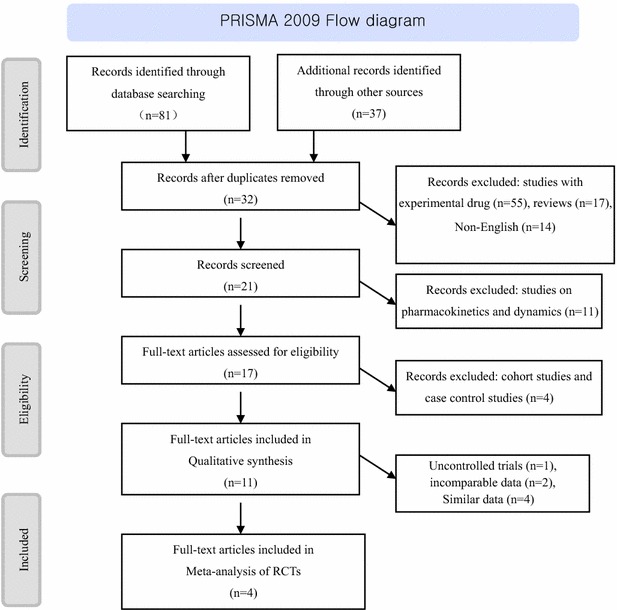
Literature search and study selection

The study flow diagram is described in Fig. 1.

### Data extraction and quality assessment

All selected articles or abstract-only reports were carefully read and analysed. We assessed the strength of evidence by using the Grading of Recommendations Assessment, Development and Evaluation (GRADE) approach [25]. The methodological quality of the included RCTs was assessed using the standard Jadad score [26] based on evaluation of randomization, blinding, and follow-up, with a maximum score of 5 points. A score of 0–2 indicates low quality, while a score of 3–5 indicates high quality. The Jadad scores of selected papres are shown in Table 1.

**Table 1:** Baseline characteristics and Jadad score of included study

Authors publish time	Study phase	Location	Sample characteristics			Trial quality			
			Age (years)	Sex: male	Race	Blinding described, appropriate	Randomization described, appropriate	Losses to follow-up described	Jadad score
Jan van Lunzen et al. 2012. [20]	Phase IIb	France, Germany, Italy, Russia, Spain, the USA	≥18 DTG: 10 mg (Mean = 32) 25 mg( Mean = 38) 50 mg (Mean = 37) EFV: 600 mg (Mean = 40)	DTG: 10 mg (42/53) 25 mg (46/51) 50 mg (45/51) EFV: 600 mg (44/50)	White: (164/205, 80 %) Black: (25/205, 12 %) Other: (16/205, 8 %)	Yes, yes	Yes, yes	Yes	5
S. Walmsley et al. 2012. [22]	Phase III	Toronto, Canada, Belgium, Spain, Italy	DTG: 50 mg (Mean = 36) EFV: 50 mg (Mean = 35)	DTG: 50 mg (378/414) EFV: 400 mg (63/419)	White: (566/833, 68 %) Black: (200/833, 24 %) Other: (67/833, 6 %)	Yes, yes	Yes, yes	Yes	5
Pedro Cahn et al. 2013. [23]	Phase III	(156 centers) Australia, Canada, Europe, Latin, America, Taiwan, South Africa, the USA	≥18 DTG: 50 mg (Mean = 42) RAL: 400 mg (Mean = 43)	DTG: 50 mg (247/354) RAL: 400 mg (238/361)	White: (347/715, 49 %) Black: (303/715, 42 %) Other: (65/715, 9 %)	Yes, yes	Yes, yes	Yes	5
Francois Raffi et al. 2013. [21]	Phase III	(100 sites) Canada, USA, Australia, Europe	≥18 DTG: 50 mg (Mean = 37) RAL: 400 mg (Mean = 35)	DTG: 50 mg (348/411) RAL: 400 mg (355/411)	White: (698/822, 85 %) Black: (88/822, 11 %) Other: (36/822, 4 %)	Yes, yes	Yes, yes	Yes	5

*DTG* dolutegravir, S/GSK1349572; *RAL* raltegravir; *EFV* efavirenz

### Data synthesis

The following data were collected: (a) basic study characteristics including study phase, single centre or multicentre; (b) population characteristics including population size, sample characteristics, pre-trial antiretroviral treatment, and exclusion criteria; (c) intervention characteristics including the drugs used, drug dosage, duration of treatment, and follow-up; (d) outcome parameters including virological and immunological responses, clinical and laboratory adverse events (AEs); (e) to compare the efficacy of DTG (INI) versus EFV (NNRTI) and RAL (INI), we performed a sub-analysis on the virological outcome. Of all studies, we selected EFV or RAL as the control drug.

### Data analysis

Two reviewers independently performed literature searching, evaluation of literature quality, information extraction, and cross checking. In case of disagreement, they discussed the issue until a consensus opinion was obtained. Statistical analyses were performed using STATA10.0 (American Computer Resource Center) and RevMan5.0 (The Cochrane Library), following the Mantel–Haenszel model to obtain weight-related risks (*p* < 0.05 for the χ^2^ statistic or a non one between-study variance by the DerSimonian and Laird [D + L] random-effects model) and 95 % confidence intervals (CIs) of virological outcome data [27, 28]. We required statistical heterogeneity of treatment effects among trials within each meta-analysis, because meta-analyses with homogenous treatment effects among trials are unlikely to find that estimates of treatment effects are associated with quality measures (or other factors).

### Sensitivity analysis

The primary results may be influenced by the use of fixed-effects models or random-effects models, as well as high-quality studies or low-quality studies. Sensitivity analysis was performed by excluding one low-quality study and one study with no statistical significance. Due to insufficient data, it is not always possible to carry out anticipated subgroup comparisons and sensitivity analyses. We could not perform a planned subgroup comparison on different corticosteroid agents, because the included studies all used DTG.

### Estimate of publication bias

Data were analysed using STATA 10.0 software. We calculated the relative risk (RR) with 95 % CI. Statistical heterogeneity was quantified using the I^2^ statistic to measure the proportion of the overall variation, and strength of the evidence was assessed using the Chi squared test [29]. In pooling the data from included trials, a fixed-effects model was applied using the method of Mantel–Haenszel (M–H) when there was no statistically significant heterogeneity. A random-effects model was employed using the method of D + L if statistically significant heterogeneity was detected. Statistical significance of the test for heterogeneity was set at I^2^ = 50 % and *p* = 0.1. When we got different results, *p* > 0.1 or *p* < 0.1 was subject to our final confirmation. We could not perform a planned subgroup comparison because the included studies all used DTG,. A funnel plot was applied to examine potential publicationbias in the meta-analysis. The fail-safe value was calculated by formula [$N_{fs0.05} = \left( {\sum {\frac{Z}{1.64}} } \right)^{2} - k$] [30].

## Results

### Selection process and study characteristics

The detailed search steps are summarised in the flow chart (Fig. 1). We initially identified 118 citations. After reading their titles and abstracts, we selected 17 potential articles for full-text view, including 11 articles of qualitative synthesis. After reading the texts, we excluded cohort studies, case control studies, uncontrolled trials, and studies with incomparable data. Finally, four articles [20–23] were related to clinical efficacy and safety RCTs of DTG, including one conference report [22].

All of the selected studies were 48 week duration, randomized, double-blind, active-controlled, non-inferiority, and multicentre trials with high scores on the Jadad scale (Table 1). All studies selected DTG-naive adults as research subjects. Of these studies, participants in two studies (50 %) were randomly assigned (1:1) to once-daily DTG 50 mg group or twice-daily DTG 400 mg group, with investigator-selected background. Subjects in other two studies (50 %) studies [20, 22] were randomly assigned to receive once-daily DTG group or 600 mg efavirenz group. Three of the studies [20–22] selected an open-label dual NRTI backbone regimen with a fixed-dose combination tablet, either abacavir/lamivudine (ABC/3TC) or tenofovir/emtricitabine (TDF/FTC). One studies [23] combined a variety of backbone regimens. These studies and the data used in the meta-analysis are listed in Table 2.

**Table 2:** Overview of studies in systematic review, grouped according to outcome data from virology and immunology response and serious drug- related AEs

Authors and publish time	Virology and immunologic response		Serious drug- related AEs	
	Experiment group, % (n/N)	Control group, n (%)	Experiment group, n (%)	Control group, n (%)
Jan van Lunzen et al. 2012. [20]	10 mg: 91 % (48/53) 25 mg: 88 % (45/51) 50 mg: 90 % (46/5150)	EFV (600 mg): 82 % (41/50)	0	2 % (1/50)
Pedro Cahn et al. 2013. [23]	50 mg: 71 % (251/354,378)	RAL (400 mg): 64 % (230/414)	1 % (2/378)	1 % (4/414)
Francois Raffi et al. 2013. [21]	50 mg: 88 % (361/411)	RAL (400 mg): 85 % (351/411)	<1 % (3/411)	1 % (5/411)
S. Walmsley et al. 2013. [22]	50 mg: 88 % (364/414)	RAL (400 mg): 81 % (338/419)	<1 % (1/414)	2 % (8/419)

*DTG* dolutegravir, S/GSK1349572; *RAL* raltegravir; *EFV* efavirenz

### Meta-analysis

Subsequently, a meta-analysis of virological outcome (number of patients achieving HIV RNA <50 copies/mL) was performed on the four controlled studies that compared a DTG-based regimen with EFV or RAL for similar indications, in which the same endpoints could be evaluated (results available for the same measures and the same time points). Secondary endpoints analysed were the changes from baseline in CD4+ cell counts and the incidence of treatment-emergent genotypic and phenotypic resistance to DTG and other antiretroviral therapies used in the study [20–23].

### The efficacy of DTG for all different subjects

**Fig. 2:**
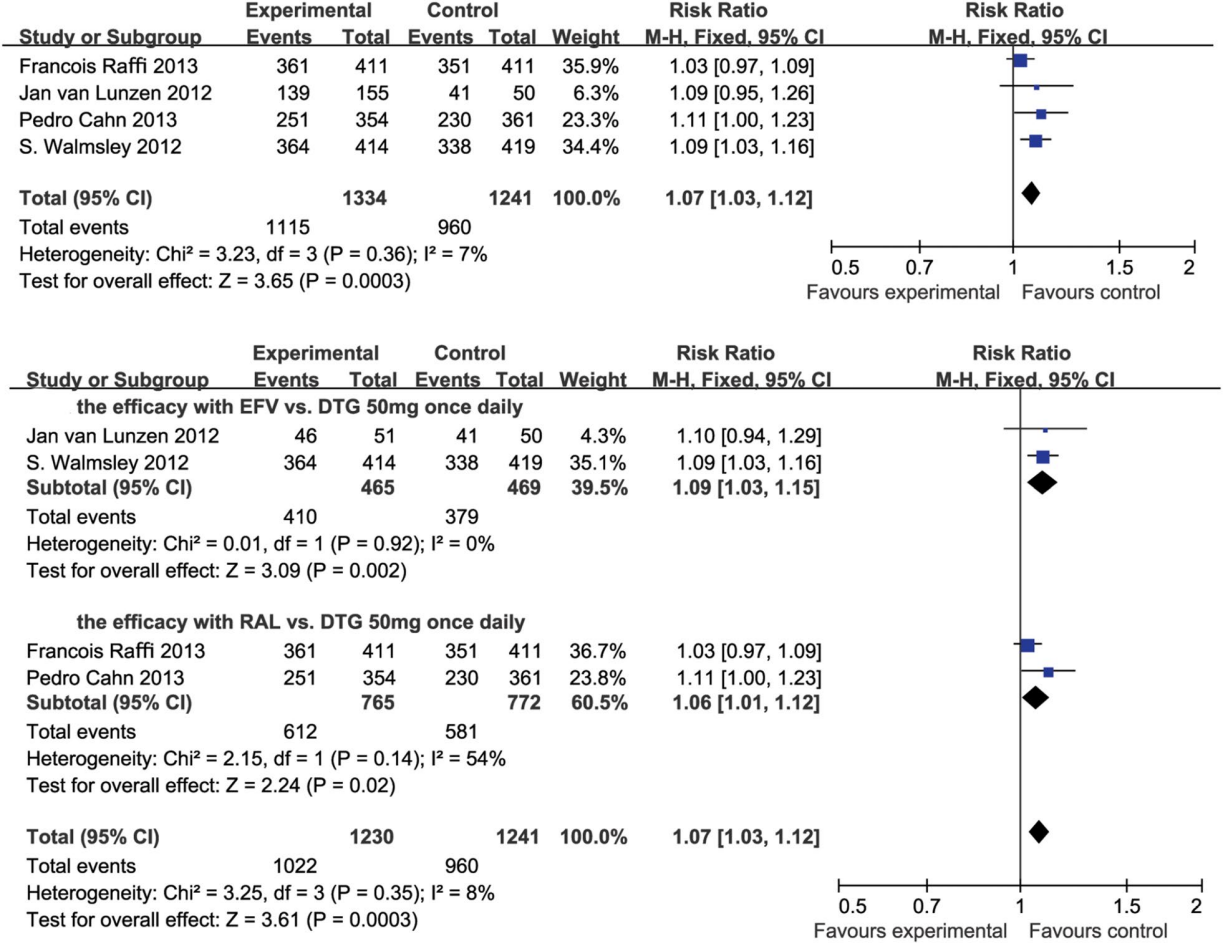
Forest plot of studies with patients switching with suppressed viral load

The four studies [20–23] included in a total of 2575 HIV-infected subjects, with 1334 experimental subjects (dose was not distinguished) and 1241 control subjects. Based on our pre-defined criteria for meta-analysis, DTG-based regimens showed a better virological outcome, which got a significant difference in the intention-to-treat (ITT**)** meta-analysis (RR 1.07, 95 % CI 1.03–1.12, *p* = 0.0003, I^2^ = 7 %; Fig. 2).

Median CD4+ cell counts increased compared to baseline level in all studies, and DTG regimens had a higher level of CD4+ cell counts than traditional antiretroviral drugs (EFV or RAL) [20–23].

#### The efficacy of DTG 50 mg once daily

All of the studies [20–23] contained a 50 mg once-daily subgroup in the experimental group, so we selected this subgroup to analyse. There were 1022 subjects in the experimental group and 960 subjects in the control group. The results of the ITT meta-analysis were RR 1.07 (95 % CI 1.03–1.12), *p* = 0.0003, I^2^ = 8 % (Fig. 2).

#### The efficacy of DTG 50 mg once daily vs. EFV and RAL

In these studies, 379 subjects received EFV in the control group and 410 subjects received DTG reversely [20, 22]. The Forest plots of the meta-analysis are shown in Fig. 2. The results were statistically significant (RR 1.09, 95 % CI 1.03–1.15, *p* = 0.002, I^2^ = 0 %).

There were 772 subjects who received RAL and 765 subjects received DTG in the experimental group [21, 23]. The Forest plots of the meta-analysis are shown in Fig. 2. The outcome of subjects who received RAL 400 mg twice daily was ITT RR, M-H, fixed (95 % CI) 1.06 (1.01–1.12), Z = 2.23 (*p* = 0.02), I^2^ = 54 % (*p* = 0.14).

### The safety of DTG for different subjects

**Fig. 3:**
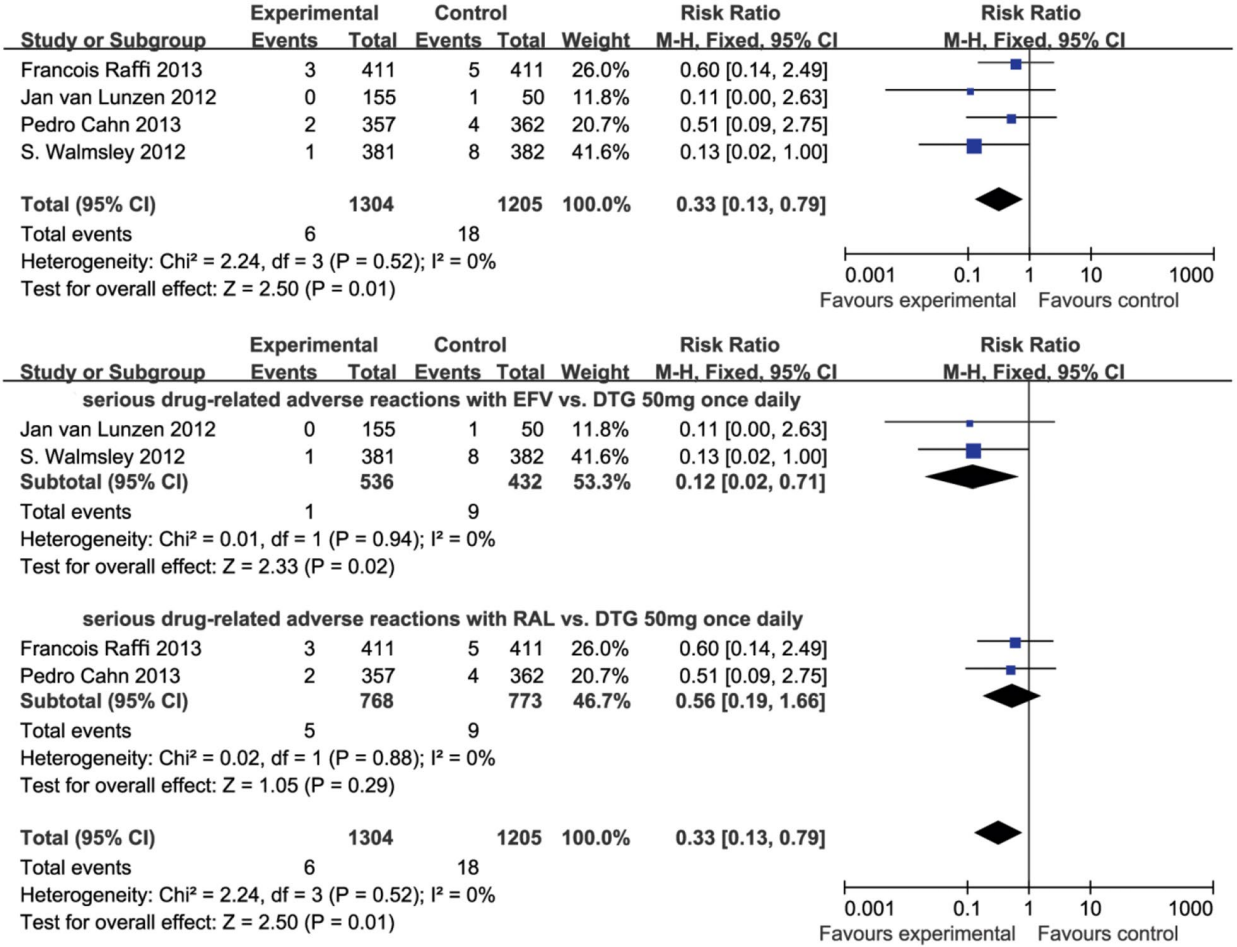
Forest plot of studies with patients switching with serious drug-related AEs

Three of the selected studies [20–22] had a larger proportion of participants, including 980 subjects in the experimental group and 880 subjects in the control group. An ITT analysis of the serious drug-related adverse reaction at 48 weeks comparing DTG versus atazanavir(ATV**)** showed an RR favouring DTG over efavirenz/raltegravir (EFV/RAL**)** in the meta-analysis (ITT, RR 0.33, 95 % CI 0.13–0.79, *p* = 0.25, I^2^ = 0 %; Fig. 3). Furthermore, when the DTG 50 mg once daily group and EFV/RAL group were analysed, the results were similar: RR 0.33, 95 % CI 0.13–0.79, *p* = 0.25, I^2^ = 0 % (Fig. 3).

#### Serious drug-related adverse reactions with DTG 50 mg once daily vs. EFV and RAL

In these studies, there were 432 subjects who received EFV in the control group and 536 subjects who received DTG reversely [20, 22]. The results included a study of comparing DTG with EFV (RR 0.12, 95 % CI 0.02–0.71, *p* = 0.02, I^2^ = 0 %). The Forest plots of the meta-analysis are shown in Fig. 3.

There were 773 subjects who received RAL and 768 subjects who received DTG in the experimental group [21, 23]. Three studies compared DTG with RAL (RR 0.56, 95 % CI 0.19–1.66, *p* = 0.29, I^2^ = 0 %). The Forest plots of the meta-analysis are shown in Fig. 3.

### Sensitivity analysis

The primary results were not influenced by the use of fixed-effects models compared with random-effects models, or high-quality studies compared with low-quality studies.

### Publication bias

The included studies appeared in the funnel plot completely and were distributed around the pooled RR, with large sample size at the top. We also performed funnel chart linear regression analysis. The results on efficacy showed that the intercept’s 95 % CI −5.144186 to 8.272042, contained 0; *p* = 0.421 > 0.1, and the results of the 50 mg group showed that the intercept’s 95 % CI −4.91207 to 7.845071, containing 0, *p* = 0.427. This indicated that the funnel plot was symmetrical. In addition, the results on safety showed that the 95 % CI −85.60035 to 74.68191, containing 0, *p* = 0.546 > 0.1.

The fail-safe value of this study was 15.11 and 54.56, which means that it would take at least 15 and 54 unpublished, especially negative results in the literature to override the conclusion of the meta-analysis.

## Discussion

Dolutegravir (DTG, S/GSK1349572) works primarily by inhibiting the enzymatic activity of HIV-1 integrase, which catalyses the insertion of viral DNA into the chromosomes of infected CD4+ lymphocytes [13, 31]. Separate trial comparing DTG-based therapy with other approved regimens could not confirm the advantages or disadvantages of DTG-based therapy. We performed a systematic review on all published clinical data concerning the integrase inhibitor DTG and subsequently carried out a meta-analysis on the virological outcome of those studies. The endpoint of the trial was the proportion of participants in each treatment group that achieved a viral load lower than 50 copies/mL at week 48, which was established by the US Food and Drug Administration, to define the time to loss of virological response (TLOVR) algorithm. Based on the meta-analysis, treatment with DTG in combination with an active background drug showed superior virological control compared with RAL 400 mg or EFV 600 mg given twice daily, although the significance was marginal. Sensitivity analysis could not identify a study that changed the results. The funnel plot was also symmetrical. Sub-analysis of subjects given DTG 50 mg once daily showed that they were more likely to achieve virological control than subjects given DTG 10 mg, 25 mg once daily. This result supported the strategy of a phase 3 clinical study to select the dose of 50 mg once daily for the study [20]. The antiviral response of DTG may be related to the favourable CD4+ T cell responses [20], and DTG may have a higher barrier to drug resistance.

The sub-analysis comparing DTG given 50 mg once daily with EFV given 600 mg given once daily showed that better viral suppression of DTG combined with dual NRTIs over EFV-based first-line therapy, which is consistent with a previous report showing that the increase of CD4+ T cells from baseline to week 48 was higher in DTG recipients than in EFV controls. Moreover, no resistance to DTG and NIRT therapy was described in the DTG group, whereas one case of NRIT and four cases of non-NRTI resistance mutation were found in EFV-based first-line therapy [20, 22]. Furthermore, improvements in viral suppression were statistically significant for DTG once-daily versus RAL twice-daily regimens in switch studies. The reason for this result appears not to be related to the marginal and nonsignificant increase in CD4+ T cells, however, is possible to be due to the treatment-emergent integrase mutation induced by RAL. Previous reports have noted that no treatment-emergent integrase mutations were detected in the DTG group. But 19 cases with integrase inhibitor-associated resistance and 4 cases with NRTI mutations were detected [21, 23]. Therefore, DTG in combination with other antiretroviral drugs (ARTs**)** has a higher virological efficacy and a higher barrier to resistance compared with RAL-based therapy and EFV-based regimens.

More attention should be paid to the safety of a new drug, as it is possible to decrease patient adherence to treatments by increasing drug toxicities. Serious drug-related adverse events were chosen as our primary outcome as a measure of the frequency of both clinically important and potentially life-threatening adverse drug events. A meta-analysis was performed on RCTs, comparing serious drug-related AEs (all grades) in DTG-based regimens with those in EFV- or RAL-based regimens. We found that patients in DTG-based regimens were twice less as likely to switch regimens due to any serious drug-related AEs compared with patients in EFV-based or RAL-based therapy. The most common AEs with DTG reported from the trials were nausea and headaches [20–23]. The forest plots for the clinical AEs suggest no statistically significant difference between DTG-based regimens and EFV- or RAL-based regimens. Although I^2^ = 54 % >50 %, *p* = 0.14 > 0.1 shows that heterogeneity in this study is qualified for getting a conclusion from meta analysis, it should not be ignored, since there are still some clinical heterogeneity among the included studies. For example, the open-label dual NRTI backbone regimen with a fixed-dose combination tablet is different in some studies. In studies of Jan van Lunzen et al., Pedro Cahn et al., and Francois Raffi et al., the regimen is TDF/FTC or ABC/3TC, but in S. Walmsley’s study, it is ABC/3TC. [22]. There was no publication bias because we have removed the poor-quality literature. Subgroup analysis highlighted some differences in outcomes reported by different studies. Subjects receiving DTG 50 mg once daily were reported no significant increase in the frequency of serious drug-related AEs compared with subjects receiving EFV 600 mg twice daily or RAL 400 mg once daily. The results consistently support the strategy of a phase 3 clinical study to select the dose of 50 mg once daily for the study [20]. An additional sub-analysis was performed to compare serious drug-related AEs in the DTG 50 mg once daily regimen with EFV-based or RAL-based regimens. The forest plot shows fewer serious drug-related AEs in DTG treatment compared with EFV-based first-line therapy. The serious drug-related AEs included hypersensitivity, psychosis, cerebrovascular accidents, renal failure, and suicide attempts. However, there was a similar rate of serious drug-related AEs including aphasia, arrhythmia, convulsion, diarrhoea, hepatitis, hypersensitivity, and phosphokinase (CPK**)** among DTG-based and RAL-based regimens, possibly due to the similar structure of these INIs.

Laboratory toxicity was estimated to assess the safety of DTG. Few subjects had an increase in alanine aminotransferase (ALT), about five or more times as the upper limit of normal, which was defined as grade 3–4 laboratory toxicity [32]. However, most of them were infected with hepatitis virus. Only one case was confirmed to be a drug-induced liver injury related to DTG. Furthermore, a non-progressive increase in serum creatinine was evident and remained stable to week 48. This result supports an in vitro study showing that DTG is a potent inhibitor of human organic cation transport (OCT**)-**2 at clinically relevant concentrations. Another study on healthy participants confirmed that DTG had no significant effect on glomerular filtration rate or effective renal plasma flow [33]. None of the patients receiving DTG discontinued the therapy because of renal toxicity. The SPRING-2 study reported no clinically significant changes over time in the fasting lipid profile in either the DTG or EFV group, while it noted that participants receiving DTG had more favourable changes in lipids than those in the EFV group [21]. With the similar pharmacological mechanism with RAL, the result was supported by in vitro experiments showing little effect of RAL on cellular adipogenesis and lipolysis [34]. Thus, DTG has the potential to improve adherence in HIV-infected patients and increases the long-term tolerability of combination ART.

Our study has some limitations. On the demographic characteristics, the low proportion of non-white and female patients enrolled was not fully representative of the global HIV/AIDS epidemic. Moreover, the selected studies were not powered to rule out all potential differences in safety, so that we could not estimate the safety of DTG-based regimen in general. Other variables might explain the relationship between the changes in CD4+ cell counts from baseline and viral load. But they were not adequately reported by the studies, including the range of changes. The effects on viral outcome and AE frequency might also be different with longer follow-up periods. But the selected trials were ongoing and data at week 96 have not been reported so far. However, we reviewed the safety and efficacy of DTG-related regimens at week 48. The time point used to calculate the proportion of patients with HIV-1 viral load less than 50 copies/mL, was the primary endpoint. Although the literature included in this analysis were of high quality, the number of studies was still small, which might reduce the stability of the conclusions.

Taken together, in this study, we found that DTG in combination with up to two additional ARTs has higher virological suppression efficacy and a higher barrier to resistance compared with RAL- or EFV-based regimen. It is also an interesting agent with the potential to improve adherence in HIV-infected patients and increase the long-term tolerability of combination ART.

## Conclusion

These results show that DTG 50 mg given once daily combined with an active background drug provides superior virological control and fewer adverse reactions compared with raltegravir 400 mg or efavirenz 600 mg given twice daily.

## Abbreviations

AIDS: acquired immune deficiency syndrome
HIV: human immunodeficiency virus
DTG: dolutegravir, S/GSK1349572
ART: antiretroviral therapy
INIs: integrase inhibitors
RAL: raltegravir
EFV: efavirenz
EVG: elvitegravir
mITT: intention-to-treat
OT: on-treatment
AT: as-treated
ABC/3TC: abacavir/lamivudine
TDF/FTC: tenofovir/emtricitabine
RR: relative risk
95 % CI: 95 % confidence interval
AEs: adverse events
NRTIs: non-nucleoside reverse transcriptase inhibitors
GRADE: Grading of Recommendations Assessment, Development and Evaluation
ATV: atazanavir
TLOVR: time to loss of virological response
ALT: alanine aminotransferase
